# Recombinant human growth hormone and insulin-like growth factor-1 do not affect mitochondrial derived highly reactive oxygen species production in peripheral blood mononuclear cells under conditions of substrate saturation in-vitro

**DOI:** 10.1186/s12986-016-0105-y

**Published:** 2016-07-04

**Authors:** James Keane, Lotti Tajouri, Bon Gray

**Affiliations:** Faculty of Health Sciences and Medicine, Bond University, Gold Coast, Queensland Australia

**Keywords:** Growth hormone, Insulin-like growth factor-1, Mitochondria, Respiratory conditions, Membrane potential, Highly reactive oxygen species

## Abstract

**Background:**

The purpose of this study was to investigate the mitochondrial effects exerted by physiological and supra-physiological concentrations of recombinant human growth hormone (rhGH) and recombinant insulin-like growth factor-1 (rIGF-1) under conditions of substrate saturation in peripheral blood mononuclear cells (PBMCs).

**Methods:**

PBMCs from healthy male subjects were treated with either rhGH, at concentrations of 0.5, 5 and 50 μg/L, or rIGF-1 at concentrations of 100, 300 and 500 μg/L for 4 h. Mitochondrial membrane potential (Δψ_m_) and mitochondrial levels of highly reactive oxygen species (hROS) were subsequently analysed. This analysis was performed by flow cytometry in digitonin permeabilized cells, following treatment with saturating concentrations of various respiratory substrate combinations and the use of specific electron transport chain (ETC.) complex inhibitors, enabling control over both the sites of electron entry into the ETC. at complexes I and II and the entry of electrons from reduced carriers involved in β-oxidation at the level of ubiquinol.

**Results:**

Neither rhGH nor rIGF-1 exerted any significant effect on Δψ_m_ or the rate of hROS production in either lymphocyte or monocyte sub-populations under any of the respiratory conditions analysed.

**Conclusion:**

That neither hormone was capable of attenuating levels of oxidative stress mediated via either complex I linked respiration or lipid-derived respiration could have serious health implications for the use of rhGH in healthy individuals, which is frequently associated with significant increases in the bioavailability of free fatty acids (FFA). Such elevated supplies of lipid-derived substrates to the mitochondria could lead to oxidative damage which would negatively impact mitochondrial function.

## Background

The electron transport chain (ETC.) is the principal site of intracellular reactive oxygen species (ROS) production, with 1–3 % of electrons transferred to O_2_ in a single reduction reaction creating superoxide (O_2_^−^) under normal physiological conditions [1–3]. This in turn leads to the formation of hydrogen peroxide (H_2_O_2_) through the actions of superoxide dismutase [4]. Hyper-polarisation of mitochondrial membrane potential (Δψ_m_) or inhibition of electron flow through the ETC. leads to a prolonged reduction of core electron transferring components within the enzyme complexes, resulting in an increased rate of O_2_^−^ production [3]. It is widely recognised that O_2_^−^ production is derived from electron ‘leakage’ from both NADH dehydrogenase (Complex I) and cytochrome c-oxidoreductase (Complex III) [5]. However, studies attempting to identify the site at which the highest rates of O_2_^−^ are generated have yielded conflicting results [5, 6]. Additionally, enzymes involved in the transfer of electrons during β-oxidation, such as electron transfer flavoprotein (ETF) and electron transfer flavoprotein quinine oxidoreductase (ETF-QO), have also been identified as potential sources of O_2_^−^ generation within the ETC. [6].

Accumulation of O_2_^−^ and H_2_O_2_, beyond the capacity of the cell’s antioxidant defences to neutralize these molecules, results in the generation of highly reactive and short lived intermediates, such as peroxynitrate (ONOO-) and hydroxyl radical [OH(•)] [1, 7]. These intermediates, termed “highly reactive oxygen species” (hROS), are generated by the reaction of O_2_^−^ with nitric oxide (NO) and of H_2_O_2_ with ferrous iron (Fe^2+^) respectively. While direct tissue damage has only been attributed to O_2_^−^ and H_2_O_2_ when present at supra-physiological concentrations, oxidative stress associated with the presence of OH(•) is well established, owing to its high non-specific reactivity with a variety of biomolecules [8]. In addition, whereas O_2_^−^ and H_2_O_2_ are neutralized through the activity of antioxidant enzymes, there are no known antioxidant defences responsible for the removal of hROS [8]. The subsequent oxidative damage to intracellular proteins, lipids and nucleic acids caused by hROS is implicated in the process of aging and associated with the development of several pathological conditions including atherosclerosis, hypertension, cancer, diabetes and Parkinson’s disease [2, 9, 10].

A decline in the systemic release of growth hormone (GH) and its subsequent stimulation of insulin-like growth factor-1 (IGF-1) production is a well-known effect of aging in both humans and experimental animals [4, 11]. Indeed, it has been demonstrated that, at circulating concentrations of approximately 5–10 μg/L, these hormones play an important role in modifying intracellular levels of oxidative stress [12–14]. Csiszar et al. [4] found that incubation of human coronary arterial endothelial cells (HCAECs) for 24 h in the presence of recombinant IGF-1 (rIGF-1) (10–1000 μg/L) and recombinant human growth hormone (rhGH) (333–3333 μg/L) significantly reduced the mitochondrial production of O_2_^−^ as well as overall O_2_^−^ and H_2_O_2_ cellular concentration levels. Of note, previous research from our laboratory has demonstrated that four hours in the presence of rhGH, at physiological concentrations (5–10 μg/L), was enough to significantly reduce production rates of mitochondrial O_2_^−^ molecules in peripheral blood mononuclear cells (PBMCs) [15]. Several authors have also noted that GH is physically capable of translocating to mitochondria [16, 17]. Ardail et al. [17] observed that, in isolated mitochondria, succinate dehydrogenase (SDH) (Complex II) and cytochrome c oxidase (COX) (Complex IV) exhibited decreased levels of activity in the presence of high concentrations of rhGH (2200 μg/L). This would indicate that the hormone can attenuate mitochondrial O_2_^−^ generation via direct modulation of the Δψ_m_.

Despite the documented antioxidant effects resulting from these hormones, at supra-physiological concentrations in-vivo GH is associated with adverse effects leading to negative implications for normal physiological functions [18, 19]. Patients with acromegaly, a pathological disorder associated with a hyper-secretion of GH from pituitary adenomas, suffer from a plethora of complications involving neurological, cardiovascular, respiratory and metabolic processes. This condition leads to increased mortality of these patients if GH and IGF-1 concentrations are not controlled and reduced to normal levels [20]. Interestingly, the prevalence of diabetes in acromegalic patients ranges between 20 and 46 %. Additionally, in several studies the inducement of supra-physiological GH concentrations in healthy subjects has been linked to the development of insulin resistance [18–20]. This is of significance since increased levels of oxidative stress have been demonstrated to directly induce insulin resistance, while it is also widely cited as a causative factor in the development of diabetes [19, 21]. This suggests that the antioxidative capacities provided to cells by GH and IGF-1, as observed in some studies in-vitro, is negated at supra-physiological hormonal concentrations in-vivo. Studies in animals lend support to this hypothesis, with transgenic mice manipulated to over-express GH being found to have increased levels of oxidative damage to liver proteins and a suppressed antioxidant capacity when compared to wild type mice [22].

While current studies examining the effect of rhGH and rIGF-1 on cellular oxidative status have analysed levels of O_2_^−^ and H_2_O_2_ production [4, 12, 13], to date no study has looked at how these hormones affect levels of hROS such as OH(•) and ONOO-. Additionally, it is not known how physiological concentrations of these hormones influence the rate of free radical production from the principal sites of electron ‘leak’ along the ETC. or whether these rates differ at supra-physiological concentrations. In light of these points, the present study aimed to i) evaluate mitochondrial levels of hROS and ii) identify the principal complexes involved in ROS production originating from the ETC. In line with previous findings [15] we hypothesize that under the respiratory conditions analysed rhGH and rIGF-1 will regulate oxidative phosphorylation in order to meet the energy demands associated with their metabolic effects. We propose that this will be associated with an efficient coupling of energy production at physiological concentrations leading to anti-oxidative effects, while at supra-physiological concentrations a decreased efficiency of electron transport will lead to an augmented production of hROS. This study used PBMCs following incubation with either rhGH or rIGF-1 at physiological and supra-physiological concentrations. PBMCs, which consist of lymphocyte and monocyte cell sub-populations, are easily isolated from whole blood, exhibit both GH and IGF-1 cell surface receptors [23, 24] and have previously been demonstrated to incur changes in gene and protein expression in response to rhGH and rIGF-1 [25, 26]. Flow cytometric analysis was performed simultaneously to assess hROS levels and Δψ_m_ in digitonin permeabilized cells. This was carried out following treatment with saturating concentrations of various respiratory substrate combinations and in the presence of specific ETC. complex inhibitors, enabling control over the sites of electron entry into the ETC. at complexes I and II, but also over the entry of electrons from reduced carriers involved in β-oxidation at the level of ubiquinol.

## Methods

### Subjects

Ten healthy male subjects (mean ± SEM: age = 23 ± 1 year, height = 1.78 ± .02 m, body mass = 77.1 ± 1.9 kg, BMI = 24.39 ± .71 kg/m^2^) were recruited to participate in the study which was approved by the Bond University Human Research Ethics Committee (BUHREC-RO1134). All subjects were given information on the nature of the study and the associated risks involved. These were explained to them prior to providing written informed consent. Exclusion criteria included smoking, the use of therapeutic, recreational or performance enhancing drugs, including anabolic steroids and rhGH up to twelve months prior to participation in the study, a history of diabetes, cardiovascular associated disease or the use of prescription medications.

### Reagents used

Hanks balanced salt solution (HBSS), RPMI Medium 1640, carbonyl cyanide m-chlorophenylhydrazone (CCCP), 1,1′,3,3,3′,3′-hexamethylindodicarbo-cyanine iodide (DilC_1_(5)), 3′-p-hydroxyphenyl fluorescein (HPF), and sodium pyruvate were purchased from Invitrogen (Carlsbad, CA, USA). L-malic acid (malate – pH adjusted to 7.4), sodium succinate, sodium octanoate, ammonium iron (II) sulphate, H_2_O_2_, rotenone, digitonin, sucrose, potassium phosphate monobasic (KH_2_PO_4_), magnesium chloride (MgCl_2_), potassium morpholinopropane sulphonate (MOPS), adenosine diphosphate (ADP), ethylenediaminetetraacetic acid (EDTA) and bovine serum albumin (BSA) were purchased from Sigma Aldrich (St Louis, MO, USA). Phosphate buffered saline (PBS) was purchased from Kinetic (Burpengary, QLD, Australia). Ficoll-Paque PLUS was obtained from GE Healthcare (Rydalmere, NSW, Australia). Finally, rhGH was purchased from Pfizer (Sydney, NSW, Australia) and rIGF-1 was purchased from Biocore (Sydney, NSW, Australia).

### Blood sample collection and PBMC isolation

Following an overnight fast, subjects underwent a blood sample collection, with the insertion of a catheter into an antecubital vein drawing 24 mL of blood into four 6 mL lithium heparinised Vacutainers (BD, CA, USA). Collected blood was diluted in an equal volume of sterile PBS and subsequently layered over Ficoll-Paque PLUS at a ratio of 2:1. All samples were centrifuged at 450*g for 30 mins for the separation of PBMCs from whole blood. Isolated cells were washed in HBSS and their concentration was determined using a Countess automated cell counter (Invitrogen, Carlsbad, CA, USA). Finally, cells were resuspended in RPMI 1640 cell culture medium at a concentration of 1*10^6^-cells/mL.

### GH and IGF-1 treatment

Isolated PBMCs were divided into aliquots for subsequent experimentation with cells either remaining untreated or administered rhGH (at concentrations of 0.5, 5 or 50 μg/L) or rIGF-1 (at concentrations of 100, 300 or 500 μg/L). All cell samples were subsequently incubated for 4 h at 37 °C and in the presence of 5 % CO_2_.

### Treatment with respiratory substrates and inhibitors

Following the experimental procedures and treatments, plasma membranes of these cells were permeabilized for the analysis of Δψ_m_ and hROS generation under differing respiratory conditions, a method adapted from the work of Pham et al. [27]. Sample cell aliquots were treated with digitonin at a concentration of 5 μg/mL for 5 mins, washed in PBS and resuspended in a respiratory buffer (0.25 M sucrose, 2 mM KH_2_PO_4_, 5 mM MgCl_2_, 1 mM EDTA, 0.1 % BSA, 1 mM ADP and 20 mM MOPS – pH adjusted to 7.4). The subsequent administration choice of substrate combinations with these cells gives rise to understanding either complex I, complex II or fatty acid mediated respiration.

Administration of the combination of pyruvate (5 mM) and malate (5 mM) (Pyr/Mal) activates isocitrate dehydrogenase, α-ketoglutarate dehydrogenase complex and malate dehydrogenase, which reduce NAD^+^ leading to the initiation of complex I mediated respiration. Complex II is not involved under these respiratory conditions, as malate equilibrates with fumarate at concentrations above 2 mM, inhibiting the conversion of succinate to fumarate and preventing the formation of FADH_2_ [28].

Administration of succinate (10 mM), in the presence of rotenone (20 μM) (Succ/Rot), activates SDH, which reduces FAD leading to the initiation of complex II mediated respiration. Rotenone acts to inhibit the flow of electrons through complex I and to prevent accumulation of oxaloacetate, a potent inhibitor of SDH [28]. Rotenone is also necessary to prevent reverse electron transfer to complex I, which stimulates the production of ROS [28].

Administration of the medium chain fatty acid octanoate (10 mM) and malate (2 mM) (Oct/Mal) mediate the transfer of electrons to reducing equivalents via β-oxidation. The use of octanoate, the oxidation of which is not carnitine dependent, to assess respiratory activity in isolated mitochondria has been previously demonstrated [29]. Additionally, malate at these concentrations acts as a “sparker” as it is known to significantly increase the rate of β-oxidation [28, 30].

### Analysis of variables

Mitochondrial ∆Ψ was analysed by flow cytometry using the lipophilic cationic dye, DilC_1_(5). The dye accumulates in the mitochondria as a function of membrane potential at concentrations below 100nM and fluoresces at an emission maximum of 658 nm when excited at 633 nm [31]. The generation of hROS was analysed by flow cytometry using the fluorescein derivative, HPF. HPF is a highly selective ROS indicator, only emitting a fluorescent signal upon reacting with either OH(•) or ONOO- [7]. The probe fluoresces at an emission maximum of 515 nm when excited at 488 nm.

Negative control samples for Δψ_m_ were incubated at 37 °C in 5 % CO_2_ for 5 min in the presence of 50 μM of CCCP in order to dissipate Δψ_m_. Positive control samples for hROS were incubated at 37 °C in 5 % CO_2_ for 5 min in the presence of 100 μM ammonium iron (II) sulphate and 1 mM H_2_O_2_ to induce OH(•) generation via the Fenton reaction.

Aliquots from each treatment condition (1 mL) were subsequently incubated at 37 °C in 5 % CO_2_ for 15 min in the presence of DilC_1_(5) at a concentration of 10nM and HPF at a concentration of 10 μM. Following incubation, all samples were washed and resuspended in 500 μL PBS, prior to immediate analysis by flow cytometry.

### Analysis by flow cytometry

All samples were analysed using a FACS Calibur flow cytometer (BD, Australia). For the analysis of Δψ_m_, samples were excited at a wavelength of 633 nm with fluorescent emissions detected in channels using a bandpass filter with a range of ≥650 nm. For the analysis of hROS production, samples were excited at a wavelength of 488 nm with fluorescent emissions detected in channels using a bandpass filter with a range of 515-545 nm. For all samples analysed, a total of 10,000 events were collected while lymphocyte and monocyte populations were gated and analysed separately. All data collected was analysed using Cellquest Pro software (BD, Australia).

### Statistical analysis

A factorial multivariate analysis of variance (MANOVA) was used to determine whether significant interactions existed between respiratory conditions and hormonal treatments for each of the analysed variables, while Bonferroni’s multiple comparisons were used for post hoc analysis between individual treatment conditions (SPSS Inc, PAWS Statistics Version 18, USA). Statistical significance was determined at an alpha level of 0.05.

## Results

### Mitochondrial membrane potential

**Fig. 1:**
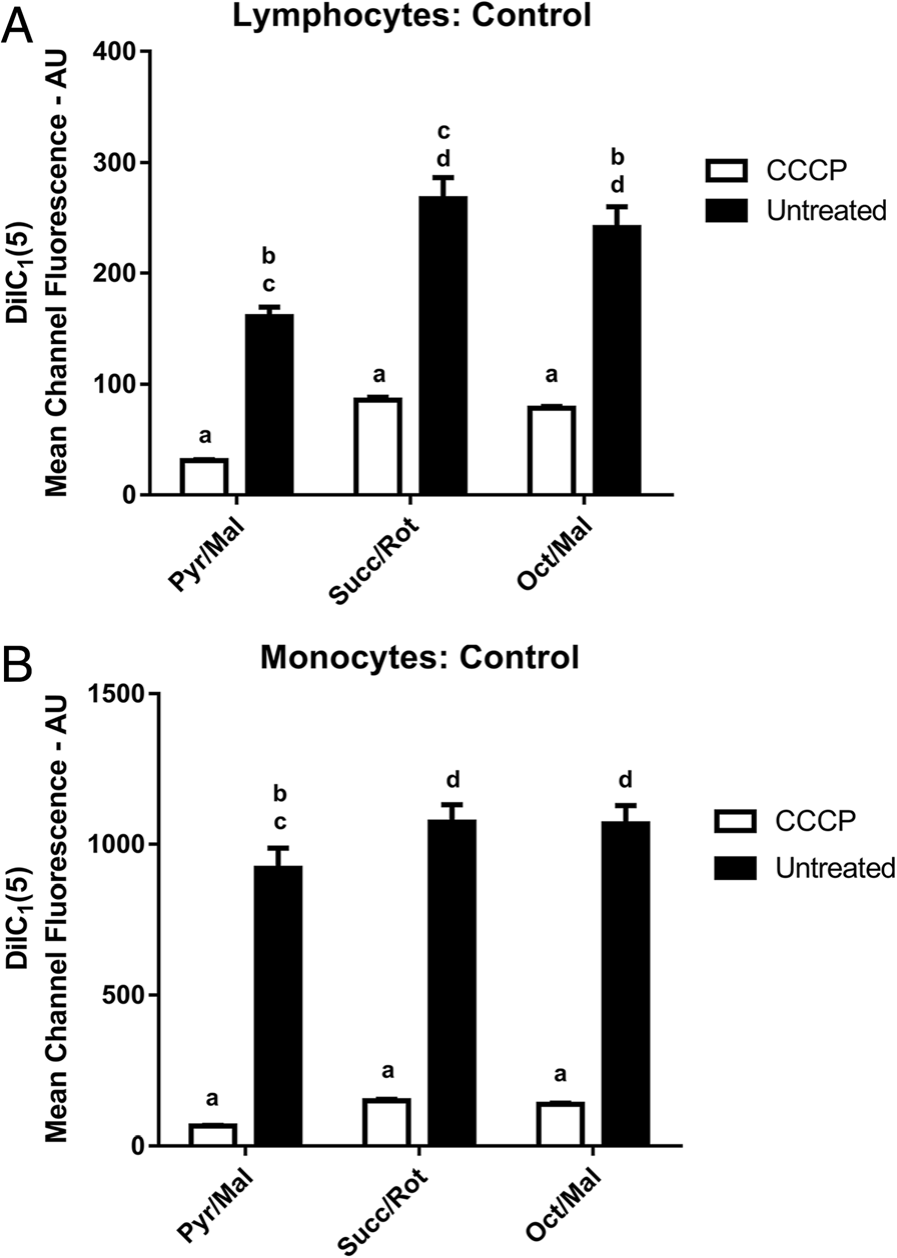
Mitochondrial Membrane Potential **a**) DilC_1_(5) Mean Channel Fluorescence from lymphocytes for negative control samples treated with CCCP compared to untreated samples under different substrate conditions. (*a* – *P* < 0.05 compared to untreated; *b* – *P* < 0.05 compared to Succ/Rot; *c* – *P* < 0.05 compared to Oct/Mal; *d* – *P* < 0.05 compared to Pyr/Mal). **b**) DilC_1_(5) Mean Channel Fluorescence from monocytes for negative control samples treated with CCCP compared to untreated samples under different substrate conditions. (*a* – *P* < 0.05 compared to untreated; *b* – *P* < 0.05 compared to Succ/Rot; *c* – *P* < 0.05 compared to Oct/Mal; *d* – *P* < 0.05 compared to Pyr/Mal)

Treatment with CCCP significantly reduced DilC_1_(5) mean channel fluorescence (AU) from samples in the presence of Pyr/Mal (mean difference ± SEM, 95 % confidence intervals, p-values: −130 ± 9 AU, −149 to -110 AU, *P* ≤ 0.05), Succ/Rot (−182 ± 19 AU, −225 to -139 AU, *P* ≤ 0.05) and Oct/Mal (−163 ± 19 AU, −205 to −120 AU, *P* ≤ 0.05) compared to untreated samples under the same respiratory conditions in the lymphocyte sub-population. Fluorescence values from Succ/Rot treated samples were significantly higher compared to Pyr/Mal (120 ± 9 AU, 96 to 144 AU, *P* ≤ 0.05) and Oct/Mal (55 ± 9 AU, 31 to 79 AU, *P* ≤ 0.05) treated samples. In addition, Oct/Mal treated samples were significantly higher compared to Pyr/Mal (65 ± 9 AU, 41 to 89 AU, *P* ≤ 0.05) treated samples (Fig. 1a).

In the monocyte sub-population treatment with CCCP also significantly reduced DilC_1_(5) mean channel fluorescence from samples in the presence of Pyr/Mal (−854 ± 67 AU, −1007 to −702 AU, *P* ≤ 0.05), Succ/Rot (−925 ± 56 AU, −1056 to −794 AU, *P* ≤ 0.05) and Oct/Mal (−930 ± 60 AU, −1067 to −794 AU, *P* ≤ 0.05) compared to untreated samples under the same respiratory conditions. Fluorescence values from Succ/Rot treated samples were significantly higher compared to Pyr/Mal (146 ± 28 AU, 70 to 222 AU, *P* ≤ 0.05) treated samples but were not significantly different from Oct/Mal treated samples (44 ± 28 AU, −31 to 120 AU, *P* = 0.73). Oct/Mal treated samples were also significantly higher in fluorescence compared to Pyr/Mal (102 ± 28 AU, 26 to 178 AU, *P* ≤ 0.05) treated samples (Fig. 1b).

**Fig. 2:**
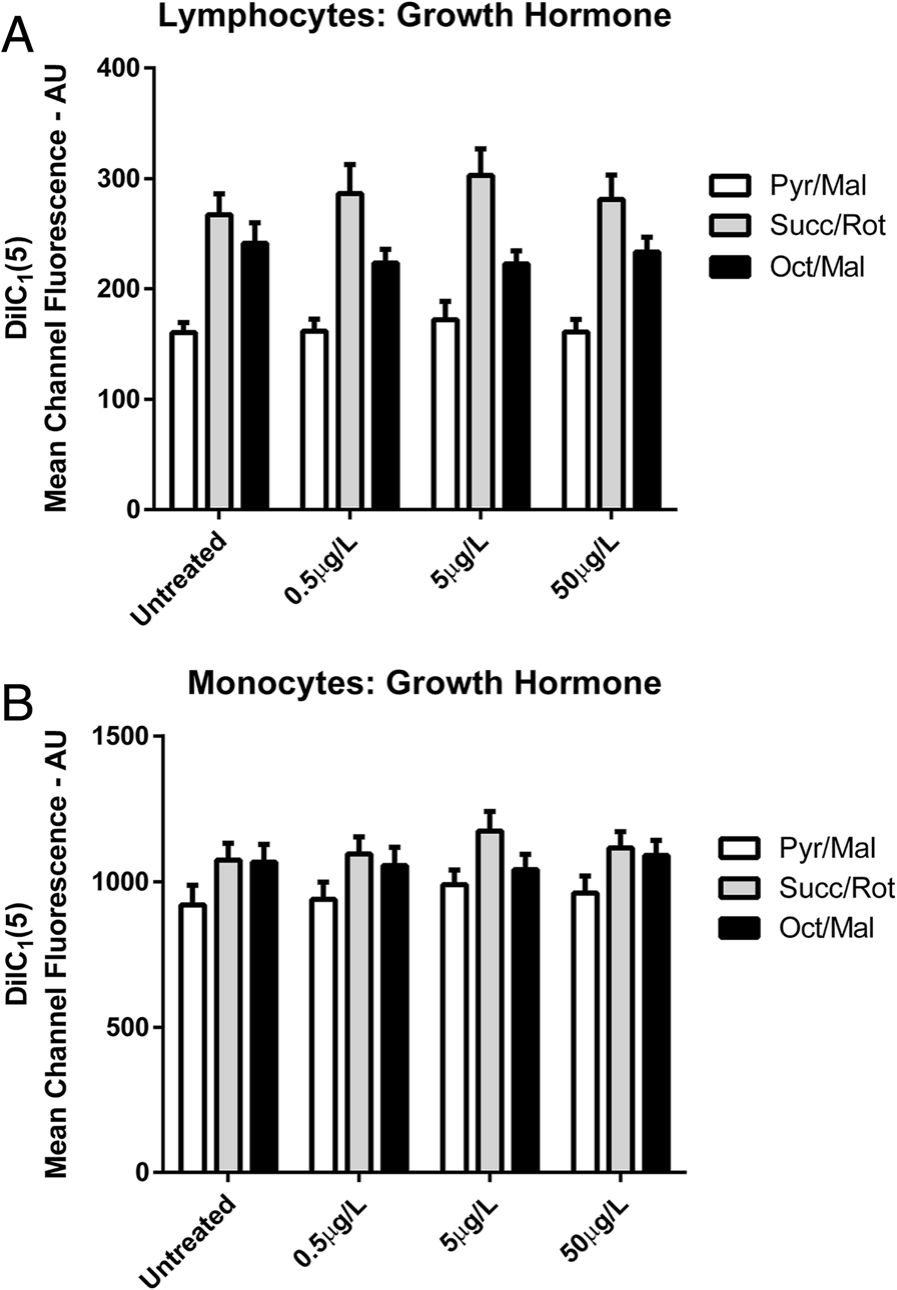
Mitochondrial Membrane Potential **a** DilC_1_(5) Mean Channel Fluorescence from lymphocytes for growth hormone treated samples compared to untreated samples under different substrate conditions. **b** DilC_1_(5) Mean Channel Fluorescence from monocytes for growth hormone treated samples compared to untreated samples under different substrate conditions

**Fig. 3:**
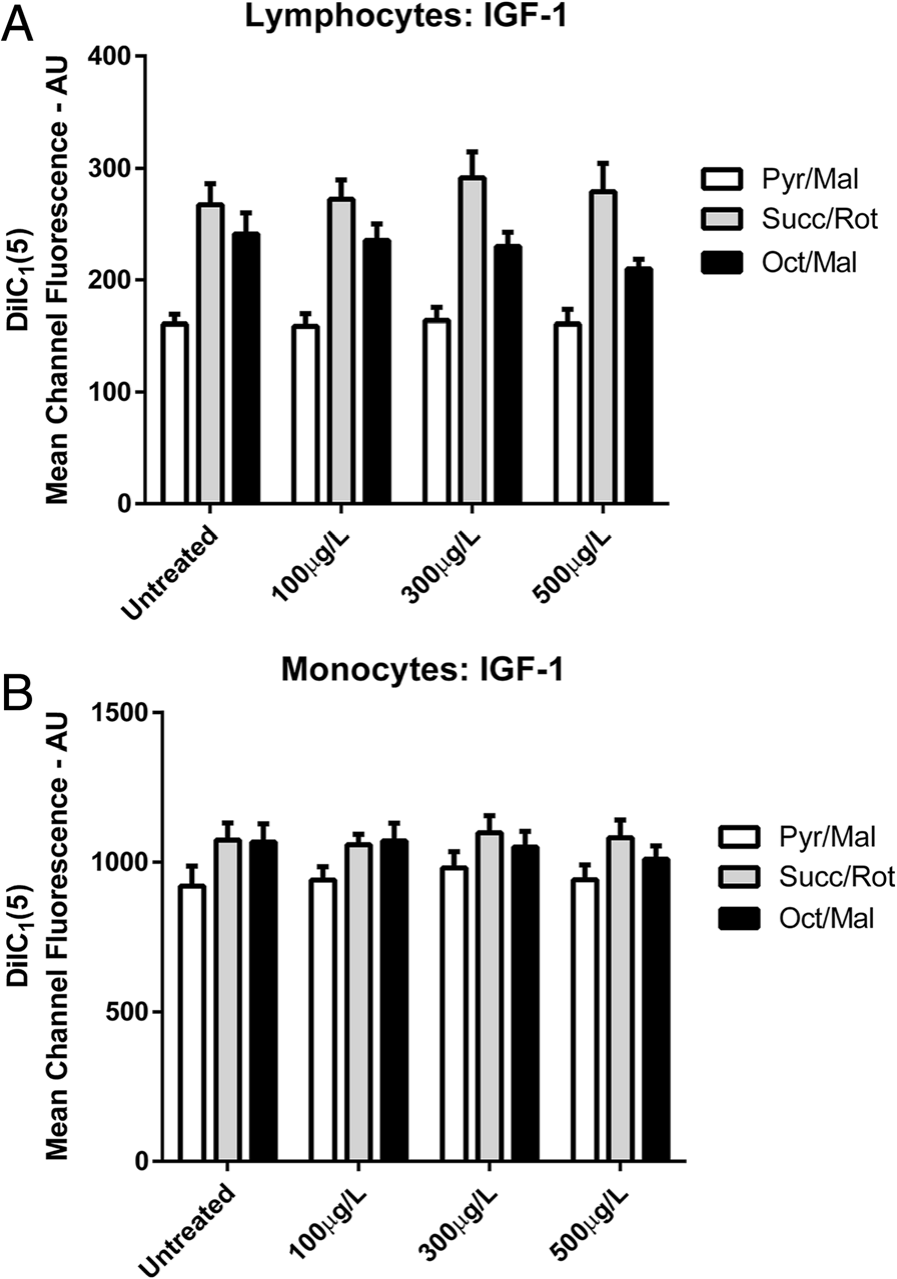
Mitochondrial Membrane Potential **a** DilC_1_(5) Mean Channel Fluorescence from lymphocytes for IGF-1 treated samples compared to untreated samples under different substrate conditions. **b** DilC_1_(5) Mean Channel Fluorescence from monocytes for IGF-1 treated samples compared to untreated samples under different substrate conditions

Neither GH (Fig. 2) nor IGF-1 (Fig. 3) exerted any significant effect on Δψm as indicated by DilC_1_(5) mean channel fluorescence values in either lymphocyte (*P* = 0.97) or monocyte (*P* = 0.75) sub-populations at any concentration administered. Finally, no significant interaction effect between hormonal treatment and respiratory substrate condition was observed in either lymphocyte (*P* = 0.99) or monocyte (*P* = 0.99) sub- populations.

### Highly reactive oxygen species production

**Fig. 4:**
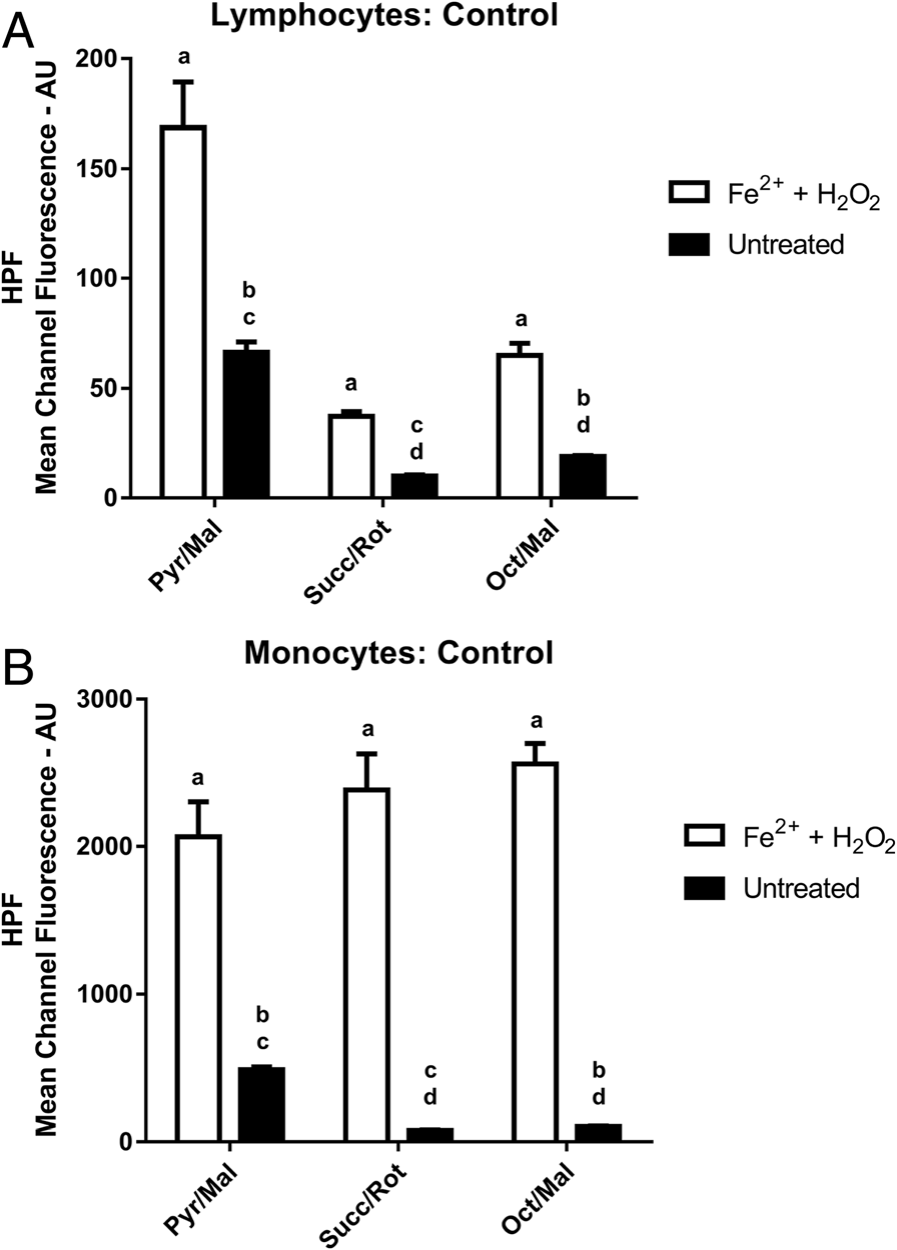
Highly Reactive Oxygen Species Production **a**) HPF Mean Channel Fluorescence from lymphocytes for positive control samples treated with H_2_O_2_ and ammonium iron (II) sulphate compared to untreated samples under different substrate conditions. (*a* – *P* < 0.05 compared to untreated; *b* – *P* < 0.05 compared to Succ/Rot; *c* – *P* < 0.05 compared to Oct/Mal; *d* – *P* < 0.05 compared to Pyr/Mal). **b**) HPF Mean Channel Fluorescence from monocytes for positive control samples treated with H_2_O_2_ and ammonium iron (II) sulphate compared to untreated samples under different substrate conditions. *a* – *P* < 0.05 compared to untreated; *b* – *P* < 0.05 compared to Succ/Rot; *c* – *P* < 0.05 compared to Oct/Mal; *d* – *P* < 0.05 compared to Pyr/Mal)

Following treatment with H_2_O_2_ and ammonium iron (II) sulphate, HPF mean channel fluorescence values (AU) were significantly higher from samples in the presence of Pyr/Mal (mean difference ± SEM, 95 % confidence intervals, p-values: 102 ± 21 AU, 55 to 149 AU, *P* ≤ 0.05), Succ/Rot (27 ± 2 AU, 22 to 32 AU, *P* ≤ 0.05) and Oct/Mal (46 ± 5 AU, 33 to 58 AU, *P* ≤ 0.05) compared to untreated samples under the same respiratory conditions in the lymphocyte sub-population. Fluorescence values from Pyr/Mal treated samples were significantly higher compared to Succ/Rot (56 ± 1 AU, 53 to 58 AU, *P* ≤ 0.05) and Oct/Mal (45 ± 1 AU, 42 to 48 AU, *P* ≤ 0.05) treated samples. In addition, fluorescence values from Oct/Mal treated samples were significantly increased compared to Succ/Rot samples (10 ± 1 AU, 8 to 13 AU, *P* ≤ 0.05) (Fig. 4a).

In the monocyte sub-population treatment with H_2_O_2_ and ammonium iron (II) sulphate also significantly increased HPF mean channel fluorescence (AU) from samples in the presence of Pyr/Mal (1578 ± 237 AU, 1042 to 2115 AU, *P* ≤ 0.05), Succ/Rot (2308 ± 246 AU, 1752 to 2864 AU, *P* ≤ 0.05) and Oct/Mal (2458 ± 137 AU, 2148 to 2769 AU, *P* ≤ 0.05) compared to untreated samples under the same respiratory conditions. Fluorescence values from Pyr/Mal treated samples were significantly increased compared to Succ/Rot (377 ± 6 AU, 360 to 393 AU, *P* ≤ 0.05) and Oct/Mal (341 ± 6 AU, 324 to 357 AU, *P* ≤ 0.05) treated samples. In addition, fluorescence values from Oct/Mal treated samples were significantly increased compared to Succ/Rot samples (36 ± 6 AU, 19 to 53 AU, *P* ≤ 0.05) (Fig. 4b).

**Fig. 5:**
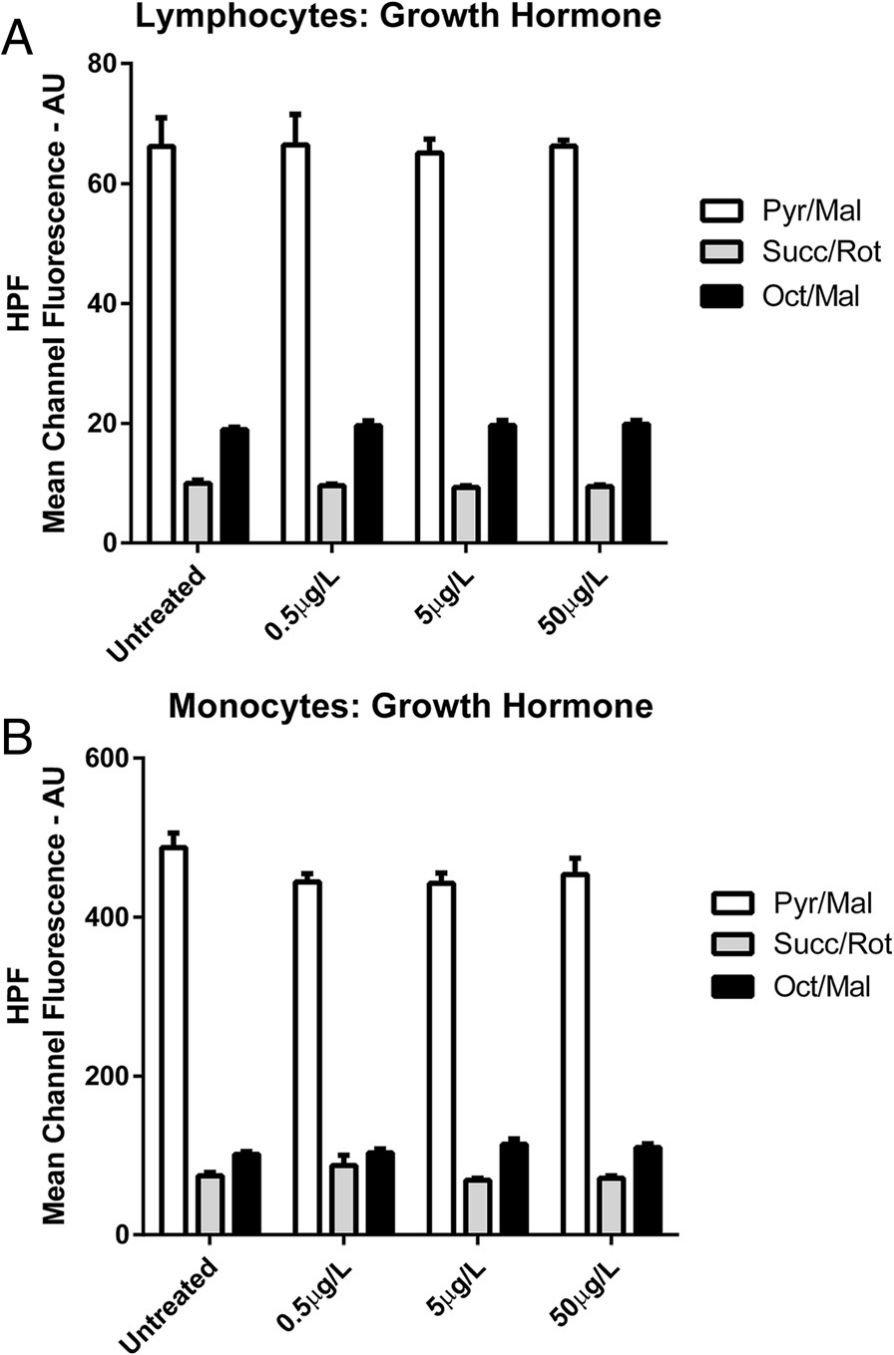
Highly Reactive Oxygen Species Production **a** HPF Mean Channel Fluorescence from lymphocytes for growth hormone treated samples compared to untreated samples under different substrate conditions. **b** HPF Mean Channel Fluorescence from monocytes for growth hormone treated samples compared to untreated samples under different substrate conditions

**Fig. 6:**
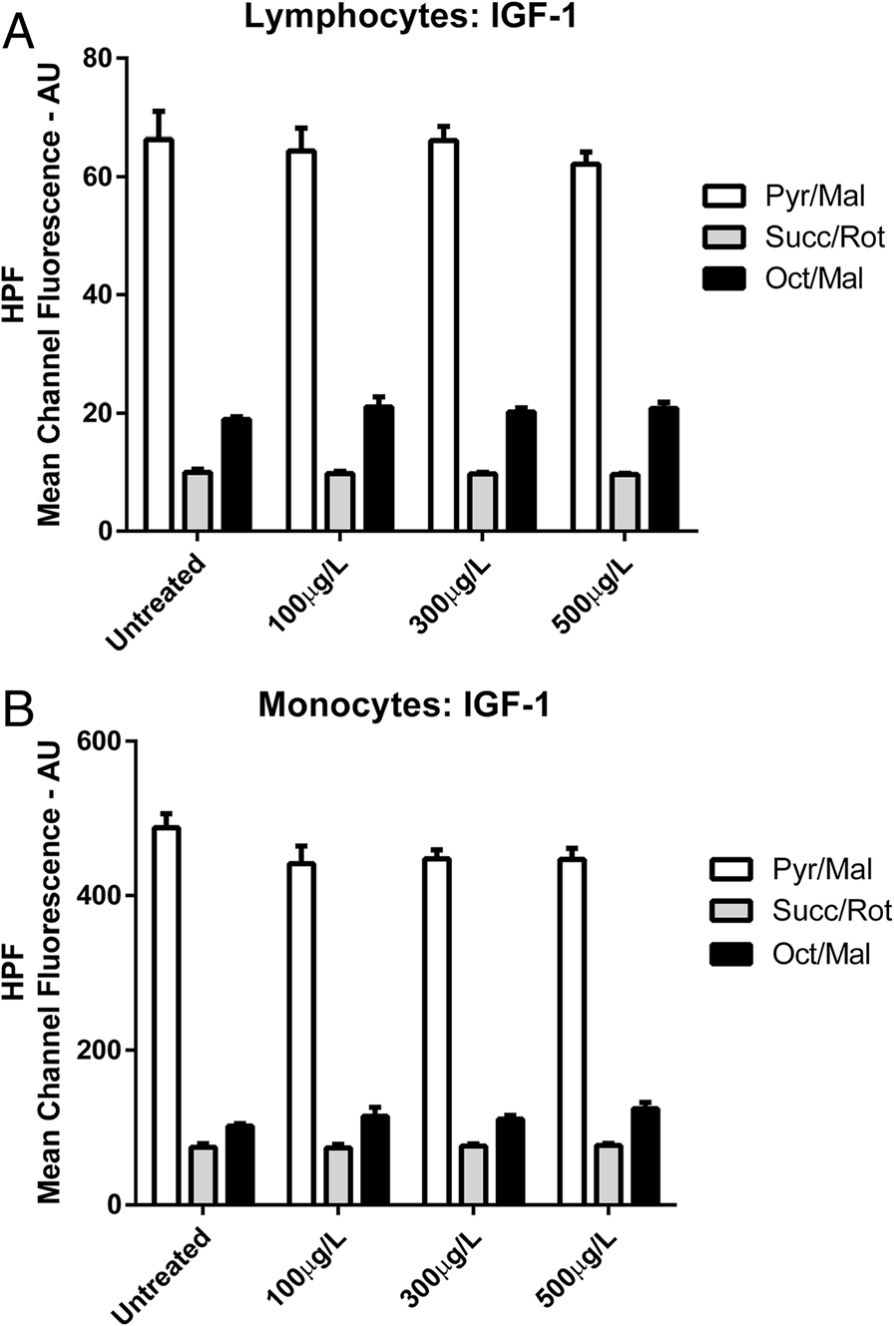
Highly Reactive Oxygen Species Production **a** HPF Mean Channel Fluorescence from lymphocytes for IGF-1 treated samples compared to untreated samples under different substrate conditions. **b** HPF Mean Channel Fluorescence from monocytes for IGF-1 treated samples compared to untreated samples under different substrate conditions

Neither GH (Fig. 5) nor IGF-1 (Fig. 6) exerted any significant effect on hROS levels as indicated by HPF mean channel fluorescence values in either lymphocyte (*P* = 0.90) or monocyte (*P* = 0.85) sub-populations at any concentration administered. Finally, no significant interaction effect between hormonal treatment and respiratory substrate condition was observed in either lymphocyte (*P* = 0.99) or monocyte (*P* = 0.39) sub-populations.

## Discussion

The principal finding of this study was that neither rhGH nor rIGF-1 exerted any significant effect on the rate of production of the highly reactive oxidants, OH(•) and ONOO-, under the respiratory conditions analysed, at any administered hormonal concentration. While this is the first study to examine the effects of rhGH/rIGF-1 on the production of these molecules, other studies have found that, in-vitro, these hormones exhibit significant effects on the generation of the reactive intermediates O_2_^−^ and H_2_O_2,_ molecules whose creation precedes that of hROS [4, 32, 33]. Csiszar et al. [4] found that both mitochondrial and cellular levels of O_2_^−^ were significantly reduced in HCAECs following treatment at both physiological and supra-physiological concentrations of rhGH (333–3333 μg/L) and rIGF-1 (10–1000 μg/L). Using the non-specific ROS probe 2′,7′-dichlorodihydrofluorescein diacetate (H_2_DCFDA), Thum et al. [32] also demonstrated significantly decreased intracellular ROS levels in cultured human endothelial cells 24 h post treatment with rhGH at concentrations of 100 and 1000 μg/L. In contrast, Gustafsson et al. [33] found that no significant change in intracellular ROS levels was induced by the presence of rIGF-1 (75 μg/L) in cultured human neuroblastoma cells under standard substrate conditions. However, rIGF-1 was effective in preventing the rise of hyperglycaemic-induced ROS production in these cells at glucose concentrations in the range of 30–60 mM. Csiszar et al. [4] related the antioxidant effects of rhGH/rIGF-1 directly to the up-regulated expression of the mitochondrial matrix antioxidant enzymes manganese-superoxide dismutase (Mn-SOD) and glutathione peroxidase-1 (GPX-1), as well as the inter membrane copper, zinc-superoxide dismutase (Cu, Zn-SOD). However, Gustafsson et al. [33] reported significant increases in uncoupling protein 3 (UCP3) expression following rIGF-1 treatment. The authors attributed the hormone anti-oxidative effects directly to a decrease in the rate of respiratory chain electron loss as a result of an uncoupling phenomenon of oxidative phosphorylation under saturating substrate conditions.

Of note, the anti-oxidative effects reported in previous in-vitro studies of rhGH and rIGF-1 are the result of a 24–72 h treatment period [4, 32, 33]. In contrast, our study was undertaken over a shorter period of four hours. It is plausible that the antioxidant effects of these hormones are time dependant and that four hours is insufficient for their manifestation. Despite this, our laboratory has previously demonstrated that human lymphocytes treated with rhGH in-vitro, at physiological concentrations (5–10 μg/L), significantly decreased levels of mitochondrial derived O_2_^−^ following only four hours of incubation. In addition, Thum et al. [32] also observed significant decreases in intracellular ROS production in human endothelial cells following GH administration after four hours, which was attributed to an improved regulation of cellular metabolic and antioxidant status. This suggests that rhGH exerts its effects on the rate of mitochondrial ROS generation via direct regulation of the organelle at the level of electron transfer within the ETC. [15]. In support of this assertion, it has previously been demonstrated that GH mediates intracellular signals that directly modulate the activity of the ETC. [17]. In the present study, neither rhGH nor rIGF-1 exerted a significant effect on Δψ_m_ under the respiratory conditions analysed at any administrated hormonal concentration. The finding that neither hormone induced a change in the rate of hROS production is possibly due to the effect exerted on Δψ_m_ by saturating concentrations of respiratory substrates. In addition, saturating concentrations of ADP were also utilized in the study in order to initiate state 3 (phosphorylating) respiration following cellular permeabilization, which doesn’t accurately reflect endogenous respiration in-vivo where cells cycle between state 3 and state 4 (non-phosphorylating) respiration [34]. Thus, any subtle effects induced by rhGH or rIGF-1 on the efficiency of oxidative phosphorylation may have been negated under the respiratory conditions analysed. Whether treatment with rhGH/rIGF-1 over longer periods (24–72 h) would exert any effect under these respiratory conditions remains undetermined and should be the target of future studies.

Sanz et al. [35] found that two weeks of GH treatment in Wistar rats resulted in significant increases in the levels of oxidative damage to mitochondrial DNA despite observed decreases in the rate of H_2_O_2_ generation, indicating that initial pro-oxidative effects preceded any changes in antioxidant capacity induced by the hormone. Together with the finding by Gustafsson et al. [33] that rIGF-1 only influenced the rate of ROS production in human neuroblastoma cells in-vitro following the addition of a stimulus to induce oxidative stress, this suggests that the changes induced by GH/IGF-1 on cellular antioxidant capacity are not mediated via a direct up-regulation of the expression of antioxidant enzymes. Indeed, it has been suggested that the influence exerted by the GH/IGF-1 axis on cellular oxidative capacity is mediated via the mechanism of mitochondrial hormesis, an adaptive response to small sub-lethal increases in mitochondrial derived ROS concentrations, which subsequently act as signalling molecules leading to an up-regulation of antioxidant enzymes and increased resistance to oxidative stress [12, 36]. In addition, there is growing evidence to suggest that Sirtuins or Sir2 (silent information regulator 2)-related enzymes (SIRT1–SIRT7) may mediate this response in relation to GH/IGF-1 [37]. The presence of oxidative insult has been found to significantly increase the expression of SIRT1, which is known to act via a deacetylation of the forkhead box O (FoxO) transcription factor to exert changes in the expression of numerous proteins that regulate mitochondrial metabolism and antioxidant capacity, among which is an up-regulation of the antioxidant enzymes, Mn-SOD and GPX-1 [12]. GH deficient Lewis Dwarf rats exhibit a significant down-regulation of SIRT1, which was found to be reversed following repletion of physiological GH concentrations [12]. SIRT4 is an ADP-ribosyltransferase which serves as a negative regulator of mitochondrial oxidative capacity [38]. The expression of SIRT4 is found to be down-regulated in obese subjects, likely in response to oxidative stress arising from greater concentrations of FFA availability [38]. In addition, a low GH/IGF-1 status was also found to be associated with a significant decrease in circulating levels of SIRT4 [39]. Such a response is mediated in an attempt to increase FFA oxidative capacity and subsequently reduce mitochondrial ROS production [38].

It is likely that modulation of oxidative phosphorylation by the GH/IGF-1 axis is not the only factor affecting the efficiency of electron transfer in-vivo. Other factors which induce oxidative stress by up-regulating the rate of mitochondrial ROS production likely contribute to an interactive effect on mitochondrial respiration which is necessary for the production of ROS at concentrations that will maximize their effect as intracellular messengers to induce an antioxidant response. Hence, a pre-existing pro-oxidative status within the cell may be necessary for GH/IGF-1 to induce any significant changes in antioxidant capacity, possibly explaining the absence of any significant effect on hROS levels, following pre-treatment with rhGH and rIGF-1 in-vitro in the present study.

In animal studies, cellular oxidative responses to rhGH and rIGF-1 administration have varied depending on the cell type analysed, indicating that any antioxidant effects of the GH/IGF-1 pathway are tissue specific [4, 35, 40]. In contrast to the findings of Csiszar et al. [4], who showed that in-vitro rhGH and rIGF-1 treatment in cardiomyocytes isolated from wild type mice significantly up-regulated the expression of Mn-SOD, Cu, Zn-SOD and GPX-1, Brown-Berg et al. [40] observed significant decreases in the expression of Mn-SOD, GPX-1 and catalase following similar treatment in murine hepatocytes. Furthermore, Sanz et al. [35] observed significant increases in oxidative damage to liver tissue isolated from Wistar rats following two weeks of GH treatment, while oxidative damage was significantly decreased in isolated cardiac tissue from the same animals. Discrepancies in the GH/IGF-1 effects between tissue types could be attributed to the expression of cell specific isoforms of GH and IGF-1 regulated locally at a tissue level [4]. Indeed, several tissue types have been shown to be capable of locally producing both GH and IGF-1, including sub-populations of human mononuclear leukocytes [41–43]. Thus, the autocrine/paracrine activation of GH/IGF-1 pathways may be more relevant to the hormonal effect on the rate of mitochondrial ROS production in these cell types, than local concentrations of the systemic isoforms secreted from the anterior pituitary and the liver, respectively [4]. In support of this, Vinciguerra et al. [44] showed that the “local muscle specific” IGF-1 isoform (mIGF-1) in murine cardiomyocytes elicited a protective effect in response to paraquat induced oxidative stress, while increasing concentrations of the systemic IGF-1 isoform itself elicited a pro-oxidative response. The mIGF-1 isoform contains a class 1 signalling peptide sequence on its N-terminal which is 48 amino acids in length and a C-terminal “Ea extension” peptide sequence containing 35 amino acids, which the mature systemic IGF-1 isoform does not possess [44–46]. Both isoforms are known to trigger phosphorylation of the IGF-1 receptor [44]. However, the systemic isoform is found to typically activate phosphoinositide 3-kinase/protein kinase B (PI3K/Akt) and mitogen-activated protein kinase (MAPK) dependent pathways, while mIGF-1 has been shown to instead activate 3-phosphoinositide-dependent kinase (PDK1) and serum- and glucocorticoid-inducible kinase-1 (SGK1) signalling molecules in cardiomyocytes, demonstrating differences in the isoforms respective signalling mechanisms downstream of surface receptor activation [44]. Hence, the activation of autocrine/paracrine pathways by locally produced isoforms may be required for significant effects on antioxidant capacity to be exhibited in the cell populations analysed here. For this reason, the investigation of how rhGH and rIGF-1 effect mitochondrial hROS production in other cell types, such as endothelial cells, should be the target of future studies.

The present study also found the level of mitochondrial hROS production to be significantly increased under conditions supporting NADH-linked respiration in comparison to all other respiratory conditions analysed, indicating that electron leakage at the site of complex I is an important contributor to the level of ROS production within the mitochondrial matrix. Such findings are in agreement with studies which have previously evaluated the rate of ROS production in isolated mitochondria [9, 47–49]. In contrast, under conditions of complex II linked respiration, hROS levels were significantly decreased in comparison to other respiratory conditions, indicating that complexes II – IV of the ETC. do not significantly contribute to electron leak on the matrix side of the inner mitochondrial matrix. Whether cytosolic levels of hROS were affected by electron leak initiated at complex III could not be determined in the present study due to the probable diffusion of such molecules out of the cell following surface membrane permeabilization.

In addition, mitochondrial hROS production was significantly increased in the presence of octanoate in comparison to complex II linked respiration, indicating that reducing equivalents derived from β-oxidation play a role in the generation of hROS. It must be noted that β-oxidation generates equal amount of NADH, transferring electrons to complex I, and FADH_2_ transferring electrons to ubiquinones on the inner mitochondrial membrane via ETF and ETF–QO [6]. Thus, the principal site of electron leak under conditions of fatty acid oxidation is yet to be determined. While rates of mitochondrial ROS production under conditions of lipid-derived respiration did not reach the high values observed under conditions of complex I linked respiration in the present study, St-Pierre et al. [6] demonstrated significant elevations in O_2_^−^ levels in isolated mitochondria from rat skeletal muscle and cardiac tissue when respiring on palmitoyl carnitine. The authors attributed this to a prolonged reduction of ETF and ETF-QO, in addition to an increase in complex I mediated electron leakage. Indeed, elevated concentrations of long-chain fatty acids in-vivo, in excess of the mitochondrial capacity to oxidize them, are known to become trapped in the mitochondrial matrix, elevating the redox state of the organelle and giving rise to the formation of highly reactive lipid peroxides [50]. Thus, long-chain fatty acids, which require active transportation across the inner mitochondrial matrix, may be more prone to inducing ROS production than the medium-chain fatty acids utilised in the present study, molecules that can enter and leave the mitochondrial matrix freely [51].

## Conclusions

In conclusion, it is worth noting that high hROS values were recorded under saturated conditions of both complex I mediated and lipid-derived respiration which neither rhGH or rIGF-1 were capable of attenuating at the diverse hormonal concentrations tested. This could have direct implications for the administration of these hormones in-vivo. Indeed, GH has been demonstrated to induce substantial elevations in serum FFA availability in healthy subjects [18, 52, 53], while both GH and IGF-1 are known to increase lipid derived oxidation in-vivo [18]. Such elevated supplies of lipid-derived substrates to the mitochondria could lead to oxidative damage which would negatively impact mitochondrial function, especially with long chain FA [54]. Different levels of fatty acid intake in subjects administered with GH or IGF-1 might also have health implications and further studies may be warranted to record diet related habits.

## Abbreviations

ADP, adenosine diphosphate; BMI, body mass index; BSA, bovine serum albumin; CCCP, carbonyl cyanide m-chlorophenylhydrazone; COX, cytochrome c oxidase; Cu, Zu SOD, copper, zinc-superoxide dismutase; DilC_1_(5), 1,1′,3,3,3′,3′-hexamethylindodicarbo-cyanine iodide; EDTA, ethylenediaminetetraacetic acid; ETC., electron transport chain; ETF, electron transfer flavoprotein; ETF-QO, electron transfer flavoprotein quinine oxidoreductase; Fe^2+^, ferrous iron; FFA, free fatty acids; FoxO, forkhead box O; GH, growth hormone; GPX-1, glutathione peroxidase-1; H_2_DCFDA, 2′,7′-dichlorodihydrofluorescein diacetate; H_2_O_2_, hydrogen peroxide; HBSS, hanks balanced salt solution; HCAECs, human coronary arterial endothelial cells; HPF, 3′-p-hydroxyphenyl fluorescein; hROS, highly reactive oxygen species; IGF-1, insulin-like growth factor-1; KH_2_PO_4_, potassium phosphate monobasic; MANOVA, multivariate analysis of variance; MAPK, mitogen-activated protein kinase; MgCl_2_, magnesium chloride; mIGF-1, local muscle specific IGF-1 isoform; Mn SOD, manganese-superoxide dismutase; MOPS, potassium morpholinopropane sulphonate; NO, nitric oxide; O_2_^−^, superoxide; OH(•), hydroxyl radical; ONOO-, peroxynitrate; PBMCs, peripheral blood mononuclear cells; PBS, phosphate buffered saline; PDK1, 3-phosphoinositide-dependent kinase; PI3K/Akt, phosphoinositide 3-kinase/protein kinase B; rhGH, recombinant human growth hormone; rIGF-1, recombinant insulin-like growth factor-1; ROS, reactive oxygen species; SDH, succinate dehydrogenase; SGK1, serum- and glucocorticoid-inducible kinase-1; Sir2, silent information regulator 2; UCP3, uncoupling protein 3; Δψ_m_, mitochondrial membrane potential
